# Short term improvement of migraine headaches during ketogenic diet: a prospective observational study in a dietician clinical setting

**DOI:** 10.1186/1129-2377-1-S14-P219

**Published:** 2013-02-21

**Authors:** C Di Lorenzo, G Coppola, G Sirianni, F Pierelli

**Affiliations:** 1 Sapienza University of Rome, Italy; 2 G.B. Bietti Foundation-IRCCS, Dept of Neurophysiology of Vision and Neurophthalmology, Rome, Italy; 3 Wellness and Dietary Medicine, Krom Genetics Institute, Rome, Italy

## Introduction

Migraine prophylaxys is an important clinical challenge, sometime complicated by side effects. Among that weight increase is one of most frequent.

## Background

Ketogenic diets (KDs), by drastic carbohydrate restriction, induces lipidic metabolism and Ketone bodies synthesis. Other than epilepsy, KDs were already suggested to be effective also in migraine (although in lack of definitive evidences) and in weight loss[1]. We have evaluated if headache and analgesics consumption improved in migraineurs self referred to a dietician, comparing followers of KD and followers of standard low-calories diet (SD).

## Methods

Migraineurs were found and enrolled in a dietician clinical setting. All clinical data were recorded before the diet initiation and, blind to neurologist, subjects were divided in two subgroups: KD and SD followers. After a one month period of diet, patients were re-evaluated for comparisons.

## Results

Headache frequency and drug consumption was reduced during the observation period, but only in KD group. Responder rates in KD group (52 subjects) were higher than 90% in terms of attack frequency and drug consumption in the month of observation, while SD group (56 subjects) has no effect.

## Conclusion

KD ameliorates headache and reduces drug consumption in migraineurs, while the SD is fully ineffective on migraine in a short term observation. Our findings support the role of KDs in migraine treatment, maybe modulated by KBs inhibitory effects on neural inflammation and cortical spreading depression [2], and enhancing brain mitochondrial metabolism [3]. Ketogenic VLCD could find a transient role in antagonize the ponderal increase, a common side effect among prophylactic migraine treatments.

## Conflict of interest

none.
